# Development of ^18^F/^11^C-Labeled Pyrrolo-Pyridine/Pyrimidine LRRK2 Selective PET Radioligands

**DOI:** 10.3390/ph18121790

**Published:** 2025-11-25

**Authors:** Sangram Nag, Vladimir Stepanov, Akihiro Takano, Ryosuke Arakawa, Marie Svedberg, Nahid Amini, Garrick Paul Smith, Gitte Mikkelsen, Thomas Jensen, Lassina Badolo, Morten Hentzer, Kenneth Vielsted Christensen, Benny Bang-Andersen, Andrea Varrone, Christer Halldin

**Affiliations:** 1 Department of Clinical Neuroscience, Centre for Psychiatry Research, Karolinska Institutet and Stockholm Health Care Services, 171 77 Stockholm, Sweden; sangram.nag@ki.se (S.N.); vladimir.stepanov@ki.se (V.S.); aktakano2002@yahoo.co.jp (A.T.); ryosuke.arakawa@ki.se (R.A.); marie.svedberg@shh.se (M.S.); andrea.varrone@ki.se (A.V.); 2 H. Lundbeck A/S, 2500 Copenhagen, Denmark; gsmith07@amgen.com (G.P.S.); gmi@lundbeck.com (G.M.); thje@lundbeck.com (T.J.); ban@lundbeck.com (B.B.-A.); 3 HUN-REN TKI, Department of Biophysics and Radiation Biology, Semmelweis University, 1094 Budapest, Hungary

**Keywords:** PET, LRRK2, radiolabeling, non-human primate, radiometabolites, in vivo

## Abstract

**Background/Objectives**: Leucine-rich repeat kinase 2 (LRRK2) is an enzyme implicated in Parkinson’s disease (PD) and a potential therapeutic target. LRRK2 PET radioligands could therefore function as imaging biomarkers for PD and as tools to measure enzyme occupancy of novel therapeutic candidates. This study aimed at developing novel radioligands for imaging using positron emission tomography (PET). Specific objectives were to synthesize fluorine-18-labeled pyrrolopyridine 1 ([^18^F]1), pyrrolo-pyrimidine 2 ([^18^F]2), as well as carbon-11-labeled pyrrolo-pyrimidine 3 ([^11^C]3), and examine their binding specificity, using in vitro autoradiography (ARG) and in vivo positron emission tomography (PET) imaging in non-human primates (NHPs). **Methods**: Radiolabeling was achieved either by classical one-step fluorine-18 nucleophilic substitution reaction or by methylation using carbon-11 methane. [^18^F]1 and [^18^F]2 were tested in NHP and human whole-hemisphere ARG experiments. PET imaging was performed in cynomolgus monkeys. Radiometabolites were measured in monkey plasma using gradient HPLC. **Results**: The results demonstrated successful radiolabeling of all three ligands. In ARG studies, both [^18^F]1 and [^18^F]2 displayed binding in brain slices from NHP and human samples. The binding of [^18^F]1 was blocked by cold Compound **1** and structurally distinct Compound **3**, but not by the structurally distinct LRRK2 inhibitor PFE-360. On the other hand, the binding of [^18^F]2 was blocked by PFE-360 in certain regions of the brain, indicating some level of specific binding to LRRK2. All three ligands showed relevant brain uptake (>3%ID), with highest uptake being observed for [^18^F]1, particularly in the thalamus. In contrast, brain uptake of [^18^F]2 and [^11^C]3 was evenly distributed across all brain regions. No blocking effect of [^18^F]1 was observed after pretreatment with the structurally distinct LRRK2 inhibitors PFE-360 (0.5 mg/kg, iv) and GEN-7915 (40 mg/kg). **Conclusions**: PET imaging indicated a low in vivo specific binding of the radioligands in the cynomolgus monkey brain, suggesting that the radioligands are not suitable for LRRK2 imaging in vivo with PET. This study emphasizes the challenges in the development of PET radioligands for imaging LRRK2 and the need for additional work to achieve this goal.

## 1. Introduction

Leucine-rich repeat kinase 2 (LRRK2) is a multifunctional protein that has a central function in neuronal signaling, inflammation, and cytoskeletal movement. Mutations of LRRK2 gene are associated with autosomal dominant or sporadic forms of Parkinson’s disease (PD) [1,2]. Some mutations are located in the kinase domain and have been linked to increased enzymatic activity of LRRK2, which contributes to the pathogenesis of Parkinson’s disease (PD). This connection has encouraged interest in developing therapeutic strategies aimed at inhibiting LRRK2 kinase activity to slow or halt disease progression [3]. Given the central role of LRRK2 in PD pathology [4], advanced neuroimaging techniques, such as PET, would be important for LRRK2 kinase inhibitor drug discovery [5]. Firstly, the development of a specific PET radioligand for LRRK2 would allow researchers and clinicians to non-invasively visualize and quantify the distribution and level of the enzyme in the living brain [6]. Secondly, a LRRK2 radioligand would be critical for assessment of LRRK2 kinase occupancy of new drug candidates in vivo. This would confirm that the drug effectively reaches and inhibits the enzyme in the brain. Thirdly, a LRRK2 radioligand could facilitate the stratification of patients based on their LRRK2 kinase activity, potentially identifying those who would benefit most from targeted therapies and finally it provides a means to monitor the longitudinal effects of treatment, helping to determine the efficacy and safety of new LRRK2 inhibitors during clinical trials [7,8]. It facilitates accurate evaluation of kinase occupancy, aids in optimal clinical trial design, and improves assessment of the efficacy of LRRK2-targeted therapy within the living organism [9,10]. Recent advancements have produced several compounds that bind to LRRK2, enabling researchers to investigate their properties as LRRK2 radioligands. Among the notable candidates are [^11^C]PF-06447475 [11], [^18^F]PF-06455943 [11,12], [^18^F]FMN3PA [13], [^11^C]GNE-1023 [14], [^18^F]FMN3PU [13], [^11^C]HG-10-102-01 [15] which all have been characterized in preclinical studies. All these compounds demonstrate in vitro binding and selectivity for LRRK2, with varying degrees of brain penetration observed in vivo, ranging from limited to good. However, achieving selective in vivo binding to LRRK2 remains a significant challenge for all radioligands developed to date. While some degree of specificity has been demonstrated through self-blocking experiments, attempts to confirm in vivo target engagement using structurally distinct LRRK2 inhibitors have not been successful. The development of brain-penetrant radioligands for kinase targets is inherently difficult, and in the case of LRRK2, this challenge is further exacerbated by its low expression levels in the brain.

**Figure 1:**
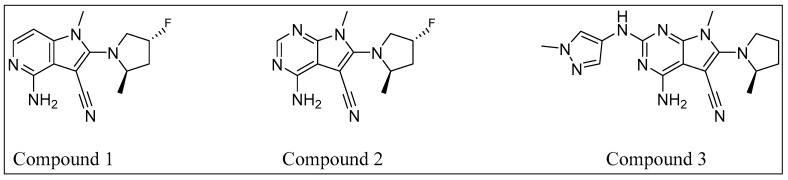
Chemical structures of Compounds **1**, **2** and **3**.

Recently, Lundbeck reported the design and optimization of a series of fragment-derived pyrrolo[2,3-d]pyrimidine-based LRRK2 inhibitors [16]. Based on the structure–activity relationship (SAR) established for this series with regard to LRRK2, binding and selectivity toward other targets, as well as key preclinical pharmacokinetic properties relevant for PET radioligands (Table 1), we selected three compounds (Compound **1**, **2**, and **3**: Figure 1) to develop radioligands for PET imaging of LRRK2 in the brain. Compounds **1** and **2** (not disclosed previously) could be radiolabeled with fluorine-18, whereas Compound **3** could be radiolabeled with carbon-11. The primary objective of this study was to radiolabel these three compounds to give specifically pyrrolo-pyridine [^18^F]1, pyrrolo-pyrimidine [^18^F]2, and pyrrolo-pyrimidine [^11^C]3. We also aimed to evaluate the binding properties of these radioligands, using in vitro autoradiography (ARG) and in vivo positron emission tomography (PET) imaging in non-human primates.

## 2. Results and Discussion

**Scheme 1:**
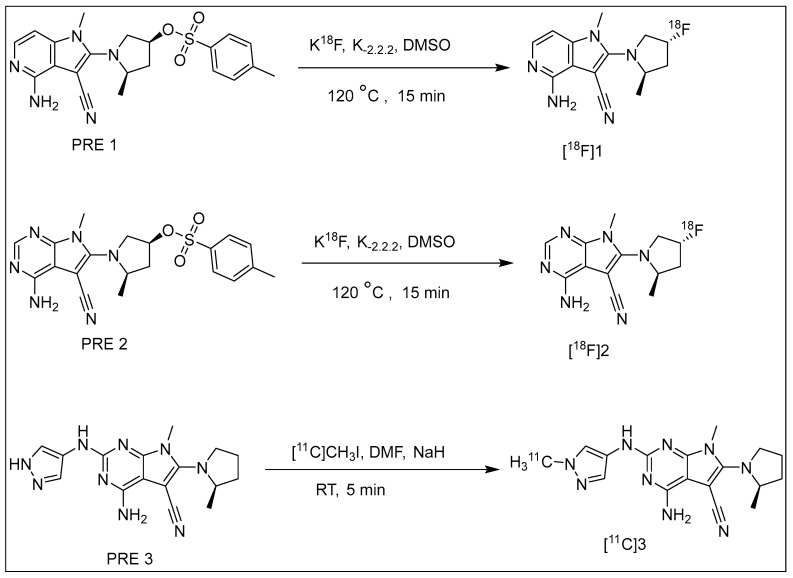
Radiosynthesis of [^18^F]1, [^18^F]2 and [^11^C]3.

The radiolabeling of [^18^F]1 and [^18^F]2 was achieved via nucleophilic substitution of their corresponding tosylate-precursors with [^18^F]fluoride in a one-pot synthesis, utilizing kryptofix K_2.2.2_ and K_2_CO_3_, as illustrated in Scheme 1. Various solvents, including DMF and DMSO, were tested at different reaction temperatures. The combination of DMSO as the solvent and a reaction temperature of 120 °C for 15 min yielded the desired radiolabeled products with the highest radiochemical yields for both [^18^F]1 and [^18^F]2. Following HPLC purification, 2.5–3.0 GBq of the final labeled products were obtained from 10 min of irradiation of cyclotron at 35 μA current. The average radiochemical yield (non-decay corrected) was >30%, with a radiochemical purity exceeding 99% for both [^18^F]1 and [^18^F]2. The entire radiosynthesis process comprising ^18^F-fluorination, HPLC purification, SPE isolation, and formulation was completed within 85–90 min. Confirmation of the radioligands’ identity was achieved by co-injection of its corresponding fluorine-19 analogue during analytical HPLC analysis, and further validation was provided by LC-MS analysis. Both [^18^F]1 and [^18^F]2 radioligands demonstrated stability in PBS buffer (pH 7.4) for 120 min. A cyclotron target produced, [^11^C]CH_4_, was used to synthesize the radiolabeling agent [^11^C]CH_3_I. A fully automated production of [^11^C]3 was performed via a single-step N-methylation reaction. Various reaction solvents, including acetone, DMF, DMSO, and acetonitrile, as well as different bases such as NaOH, KOH, and NaH, were tested. Reaction temperatures ranging from room temperature to 100 °C and varying amounts of precursor were also explored to optimize the process. The optimal conditions were achieved using [^11^C]CH_3_I as the alkylating agent and the corresponding desmethyl precursor (0.5 mg) in DMF at room temperature, with NaH as the base. The overall synthesis time of [^11^C]3 which includes HPLC purification, SPE isolation, and formulation was about 45 min. The synthesis was highly reproducible and, following cyclotron irradiation with a beam current of 35 μA for 20 min, achieved more than 1500 MBq of pure final labeled product. Formulation of the final product [^11^C]3 was completed by sterile PBS and maintained radiochemical purity exceeding 99% for 60 min.

**Figure 2:**
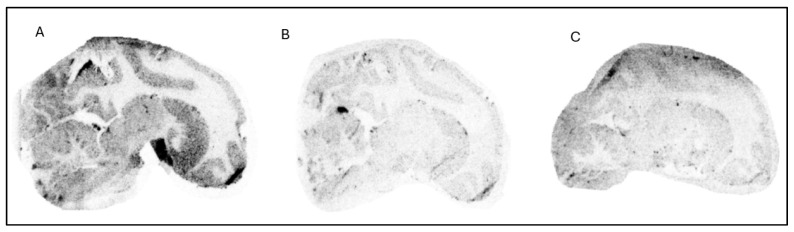
Autoradiograms of sagittal slices of a NHP brain labeled with [^18^F]1 at baseline condition (**A**) and during incubation with cold Compound **1** (10 µM) (**B**) and Compound **3** (10 µM) (**C**).

**Figure 3:**
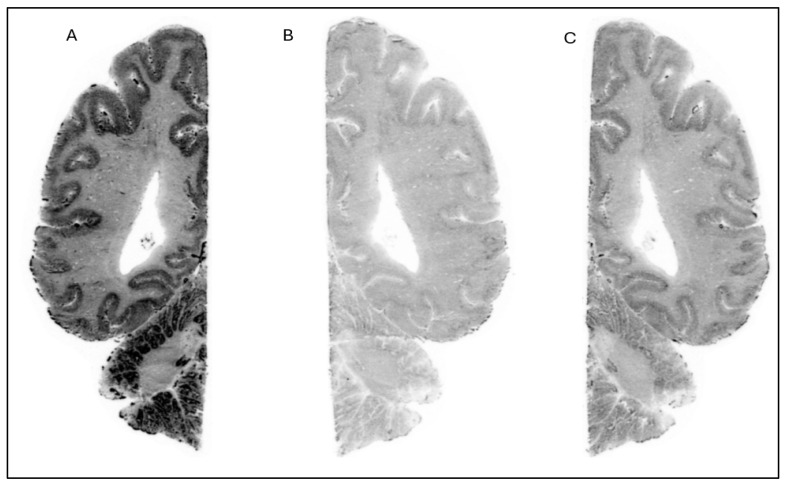
Autoradiograms of horizontal slices of a human brain labeled with [^18^F]1 at baseline condition (**A**) and during incubation with Compound **1** (10 µM) (**B**) and cold Compound **3** (10 µM) (**C**).

**Figure 4:**
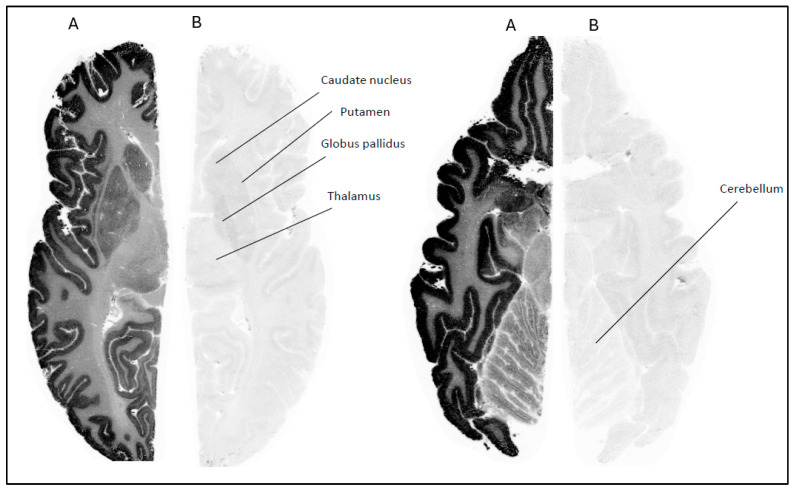
Autoradiograms of horizontal slices of a human brain labeled with [^18^F]2 at baseline condition (**A**) and during incubation with PFE-360 (10 µM) (**B**). Brain sections at the level of caudate, putamen, globus pallidus, and thalamus (**left**) and cerebellum (**right**).

The in vitro ARG studies aimed to assess the binding characteristics of the compounds; [^18^F]1 displayed clear binding in brain slices from NHP (Figure 2) and human (Figure 3). The binding was effectively blocked by an excess of cold Compound **1** (self-block) as well as Compound **3** (heterologous block), indicating that its interaction with brain tissue is likely specific. However, pre-incubation with 0.1 µM of PFE-360 [20], another structurally different LRRK2 kinase inhibitor, did not significantly reduce the binding of [^18^F]1 in a human brain slice (Figure 4). This could indicate that [^18^F]1 exhibited limited specificity but more likely that the concentration of PFE-360 used may have been suboptimal, as Compound **2** demonstrated significant blocking activity at 10 µM.

In contrast, [^18^F]2 demonstrated a different binding profile. Its binding in human brain regions such as the striatum, hippocampus, and cerebellum was significantly reduced when sections were pretreated with 10 µM of PFE-360. The displacement of [^18^F]2 by PFE-360 suggests that its binding in these regions is specific to LRRK2, making it a promising candidate for further development as an LRRK2 PET imaging agent. Overall, the differential displacement profiles underscore the importance of both tracer selectivity and binding affinity in evaluating the suitability of radioligands for neuroimaging applications. Among the tested compounds, [^18^F]2 showed promise for targeting LRRK2 in human tissues. For radioligand [^11^C]3, no ARG studies were performed. Instead, we proceeded directly to in vivo PET imaging in NHPs with all three radioligands [^18^F]1, [^18^F]2, and [^11^C]3.

**Figure 5:**
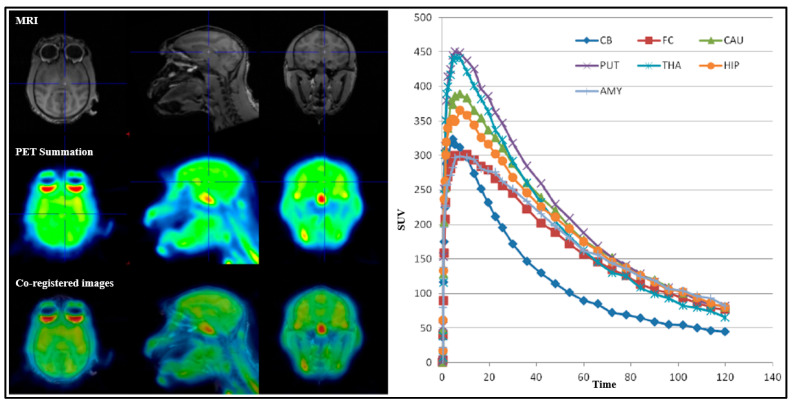
PET images (color scale is arbitrary) co-registered with MRI (**left**) and regional brain uptake of [^18^F]1 at baseline condition (**right**).

**Figure 6:**
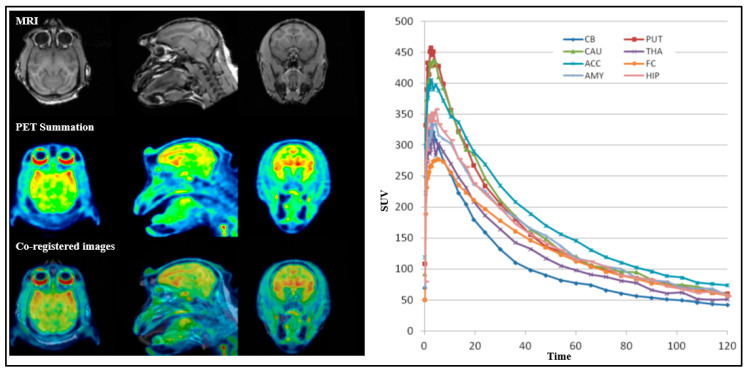
PET images (color scale is arbitrary) co-registered with MRI (**left**) and regional brain uptake of [^18^F]1 after pretreatment with GNE-7915 (40 mg/kg body weight) (**right**).

**Figure 7:**
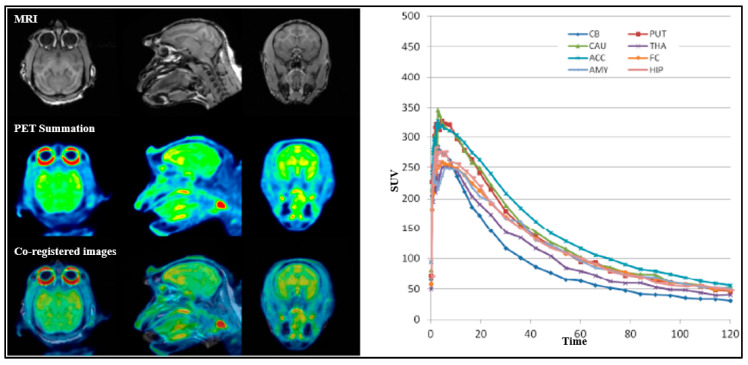
PET images (color scale is arbitrary) co-registered with MRI (**left**) and regional brain uptake of [^18^F]1 after pretreatment with PFE-360 (0.5 mg/kg body weight) (**right**).

**Figure 8:**
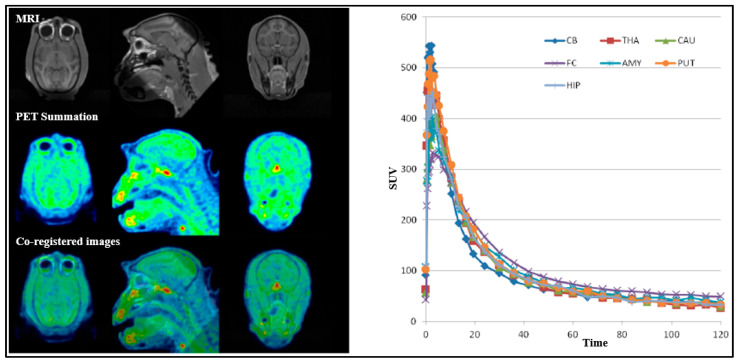
PET images (color scale is arbitrary) co-registered with MRI (**left**) and regional brain uptake of [^18^F]2 at baseline condition (**right**).

**Figure 9:**
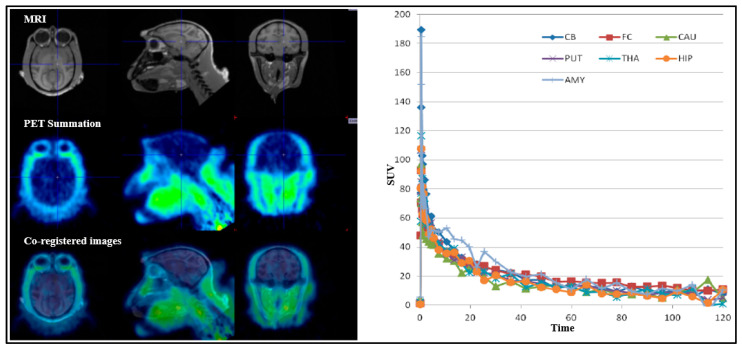
PET images (color scale is arbitrary) co-registered with MRI (**left**) and regional brain uptake of [^11^C]3 at baseline condition (**right**).

Four female cynomolgus monkeys, NHP1, NHP2, NHP3 and NHP4, were the subjects of a study involving three different radioligands, [^18^F]1, [^18^F]2 and [^11^C]3, as detailed in Table 2. A total of nine PET measurements were performed across nine separate experimental days. Seven of these measurements were conducted under baseline conditions, three with [^18^F]1, two with [^18^F]2, and two with [^11^C]3. Two additional PET measurements were conducted with [^18^F]1 following pretreatment with two different LRRK2 inhibitors, representing two distinct structural classes: GNE-7915 [21] and PFE-360. GNE-7915 (40 mg/kg body weight) and PFE-360 (0.5 mg/kg body weight) were administered 30 min prior to the injection of the radioligand [^18^F]1. [^18^F]1 demonstrated good brain uptake (>3%ID) with rapid washout, and regional differences in uptake suggest some specific binding to the target. The cerebellum showed the lowest uptake, indicating low binding in this region (Figure 5). However, no clear blocking effects were observed with GEN-7915 (Figure 6) or PFE-360 (Figure 7), indicating limited evidence of in vivo target-specific binding. Brain uptake of [^18^F]2 was high (3.8–4.4%ID at peak) with a fast washout, reaching half the peak level within approximately 20 min, and showed a relatively uniform distribution across all the brain regions (Figure 8). Conversely, [^11^C]3 exhibited moderate initial brain uptake (>1%ID) but rapid washout, with no significant regional differences (Figure 9). Due to the relatively uniform distribution and no regional differences, both [^18^F]2 and [^11^C]3 were considered inappropriate for further development. The free fraction in plasma (*f*_P_) was 45% for [^18^F]1, 60% for [^18^F]2 and 15% for [^11^C]3. For all the PET measurements with [^18^F]1, at baseline and following the administration of GEN-7915 and PFE-360, kinetic analysis was performed using 1TC and 2TCM with measurement of the arterial input function utilizing 90 min data (Table 3). The outcome measure was the total distribution volume *V*_T_ and *V*_T_/*f*_p_. No difference in *V*_T_ or *V*_T_/*f*_p_ between baseline and pretreatment was observed (Table 4). There was no effect of pretreatment on *f*_p_. The main challenge for LRRK2 PET radioligand discovery is the low Bmax of LRRK2, which requires very high-affinity compounds that are not overly lipophilic.

Radiometabolite analysis and deproteinization were performed according to previous reports [22]. Following deproteinization, more than 95% of the plasma radioactivity was successfully recovered into acetonitrile. HPLC analysis of plasma samples post-injection of three different radioligands, [^18^F]1, [^18^F]2 and [^11^C]3, showed the compounds eluting at a retention time of around 3.5 min. The parent compounds accounted for over 90% of the eluted radioactivity at 5 min, with their concentration gradually decreasing to around 10% at 90 min under baseline PET conditions. No differences were observed for [^18^F]1 after pretreatment with GEN-7915 or PFE-360. Several more polar radiometabolite peaks appeared before the parent compound eluted, indicating metabolic transformation. The identity of [^18^F]1, [^18^F]2 and [^11^C]3 was confirmed via co-injection with non-radioactive Compound **1**, Compound **2** and Compound **3**, ensuring correct peak assignment. To assess plasma protein binding, the ultrafiltration method [23] was used and corrected using control samples to account for membrane binding effects. The results showed that [^18^F]1 bound to plasma proteins at a rate of approximately 55% under baseline conditions, with no significant difference binding following pretreatment. These data suggest that a free fraction of approximately 45% of [^18^F]1 remains in plasma, regardless of pretreatment. Similarly, [^18^F]2 exhibited approximately 40% protein binding, implying that about 60% of [^18^F]2 exists in the free state. In contrast, [^11^C]3 showed high plasma protein binding, exceeding 85%, which indicates that only around 15% remains unbound in plasma.

## 3. Materials and Methods

### 3.1. General

All non-radioactive reference standards, Compound **1** (4-Amino-2-((2R,4R)-4-fluoro-2-methyl pyrrolidin-1-yl)-1-methyl-1H-pyrrolo[3,2-c]pyridine-3-carbonitrile); Compound **2** (4-Amino-6-((2R,4R)-4-fluoro-2-methylpyrrolidin-1-yl)-7-methyl-7H-pyrrolo[2,3-d]pyrimidine-5-carbo nitrile); and Compound **3** ((R)-4-amino-7-methyl-2-((1-methyl-1H-pyrazol-4-yl)amino)-6-(2-methylpyrrolidin-1-yl)-7H-pyrrolo[2,3-d]pyrimidine-5-carbonitrile), as well as the corresponding precursors, Precursor 1 ((3S,5R)-1-(4-amino-3-cyano-1-methyl-1H-pyrrolo[3,2-c]pyridin-2-yl)-5-methylpyrrolidin-3-yl 4-methylbenzenesulfonate); Precursor 2 ((3S,5R)-1-(4-amino-5-cyano-7-methyl-7H-pyrrolo[2,3-d]pyrimidin-6-yl)-5-methylpyrrolidin-3-yl 4-methyl benzenesulfonate); and Precursor 3 ((R)-2-((1H-pyrazol-4-yl)amino)-4-amino-7-methyl-6-(2-methylpyrrolidin-1-yl)-7H-pyrrolo[2,3-d]pyrimidine-5-carbonitrile), were supplied by H. Lundbeck A/S. Ottiliavej 9, 2500, Valby, Denmark. All other chemicals and reagents were sourced from commercial suppliers and utilized without further purification. Solid-phase extraction (SPE) cartridges, specifically SepPak QMA Light and SepPak C18 Plus, were purchased from Waters in Milford, MA, USA. The C18 Plus cartridges were activated by rinsing with 10 mL of ethanol followed by 10 mL of sterile water. The SepPak QMA Light cartridges were activated using a 0.5M K_2_CO_3_ solution (10 mL), followed by rinsing with 15 mL of 18 MΩ water.

Fluorine-18 fluoride ([^18^F]F) and [^11^C]methane ([^11^C]CH_4_) were produced at the Karolinska Hospital in Stockholm, Sweden using a 16.4 MeV GEMS PET trace cyclotron (GE, Uppsala, Sweden). A fully automated production was performed using a Scansys PET chemistry synthesizer (TracerMaker) supplied by Sandys Laboratorieteknik, Copenhagen, Denmark, for all radioligands. The radiolabeling processes were carried out using a custom-made semi-automated/automated synthesis module. Liquid chromatographic analysis (LC) was performed using a Merck-Hitachi gradient pump and a Merck-Hitachi L-4000 variable wavelength UV detector (Merck-Hitachi, Tokyo, Japan).

#### 3.1.1. Production of [^18^F]Fluoride ([^18^F]F^−^)

The production of ^18^F-/K_2_CO_3_/Kryptofix_2.2.2_ complex, an organic cation/inorganic anion salt, was carried out following a previously published method. For generating [^18^F]F^−^, a GEMS PETtrace cyclotron was used to bombard ^18^O-enriched water with 16.4 MeV protons through the ^18^O(p,n)^18^F reaction. The product [^18^F]F^−^ was separated from ^18^O-H_2_O utilizing a conditioned SepPak QMA, Waters, Milford, MA, USA (Quaternary Methylammonium) light anion exchange cartridge, which thereafter was washed with the solution of potassium carbonate (13 µmol, 1.8 mg), and kryptofix_2.2.2._ (4,7,13,16,21,24-hexaoxa-1,10-diazabicyclo-[8.8.8]hexacosane-K_2.2.2_) (26 µmol, 9.8 mg) in 85 µL of 18 MΩ water and 2 mL of MeCN into a reaction vessel (4 mL). The solvents were removed at 140 °C for 10 min of continuous nitrogen/helium flow at 70 mL/min to obtain a dry complex of [^18^F]F-/K_2_CO_3_/K_2.2.2_, after which the residue was cooled to room temperature.

#### 3.1.2. Synthesis of [^18^F]1 (4-Amino-2-((2R,4R)-4-[^18^F]fluoro-2-methylpyrrolidin-1-yl)-1-methyl-1H-pyrrolo[3,2-c]pyridine-3-carbonitrile) and [^18^F]2 (4-Amino-6-((2R,4R)-4-[^18^F]fluoro-2-methyl Pyrrolidin-1-yl)-7-methyl-7H-pyrrolo[2,3-d]pyrimidine-5-carbonitrile)

To the dry complex of ^18^F-F-/K_2_CO_3_/K_2.2.2_., the corresponding tosylate precursors (1 mg, 0.005 mmol) in DMSO (500 µL) were added at 120 °C and left for 15 min to produce [^18^F]1/[^18^F]2. The reaction mixture was cooled to room temperature (RT) and diluted with water (2.5 mL) to a total volume of 3 mL before being injected into a semi-preparative reverse-phase XBridge HPLC column (C18, 10 mm × 250 mm, 5 µm) for purification. The column outlet was connected to a UV absorbance detector (λ = 254 nm) in series with a GM-tube for radioactivity detection. Elution was carried out with a mobile phase consisting of CH_3_CN and ammonium formate (0.1 M) in a ratio of 24:76 at a flow rate of 6 mL/min. This process yielded a radioactive fraction corresponding to pure [^18^F]1/[^18^F]2, with a retention time (tR) of 20–22 min and collected in a bottle containing sterile water (50 mL). The resulting mixture was further purified by passing through a preconditioned SepPak SPE (Oasis HLB 3cc, Waters, Milford, MA, USA) cartridge followed by washing with sterile water (10 mL). Isolated [^18^F]1/[^18^F]2 was eluted with 1 mL of ethanol into a sterile vial containing 9 mL of sterile saline. The formulated product was then filtered through a Millipore Millex^®^ GV (Millipore, Burlington, MA, USA) sterile filter unit (0.22 μm) for further application in PET.

#### 3.1.3. Synthesis of [^11^C]3 ((R)-4-Amino-7-methyl-2-((1-[^11^C]methyl-1H-pyrazol-4-yl)amino)-6-(2-methylpyrrolidin-1-yl)-7H-pyrrolo[2,3-d]pyrimidine-5-carbonitrile)

[^11^C]Methane ([^11^C]CH_4_) was produced in-target via the ^14^N(p,α)^11^C reaction using nitrogen gas mixed with 10% hydrogen and 16.4 MeV protons generated by cyclotron. The resulting [^11^C]CH_4_ was captured into a cooled Porapak Q trap. The methyl iodide was synthesized by mixing extracted [^11^C]methane from Q traps with iodine vapor at temperatures of 60 °C before undergoing radical transformation at 720 °C in a closed-loop system. Produced [^11^C]CH_3_I was trapped in a Porapak Q trap at room temperature, and any unreacted [^11^C]CH_4_ was recirculated for three minutes. [^11^C]CH_3_I was released from the trap by heating the Porapak Q to 180 °C while flowing He gas. [^11^C]3 was obtained by trapping [^11^C]CH_3_I at room temperature in a reaction vessel containing the mixture of appropriate precursor AF67962 (1.0 mg, 3.0 mMol) and sodium hydride (3.0–5.0 mg) in DMF (500 µL). The reaction mixture was maintained at room temperature for 200 s to facilitate the reaction. After synthesis, the residue was diluted with sterile water (2 mL) and injected into the high-performance liquid chromatography (HPLC) injection loop for purification. The loop was connected to a built-in HPLC system equipped with a semi-preparative reverse-phase (RP) XBridge C18 (Waters, Milford, MA, USA) column (250 × 10 mm, 5 µm particle size). The column outlet related to a Merck Hitachi UV detector (λ = 254 nm) (VWR, International, Stockholm, Sweden) in series with a GM-tube (Carroll-Ramsey, Berkley, CA, USA) used for radioactivity detection. A mixture of acetonitrile (27%) and ammonium formate (0.1 M) (73%) with a flow rate of 6 mL/min was used as HPLC isocratic mobile phase. The radioactive fraction corresponding to the desired product [^11^C]3 was eluted with a retention time (tR) of 16–17 min and collected in a bottle containing 50 mL of sterile water. The resulting mixture was further purified by passing it through a preconditioned SepPak SPE (Oasis HLB 3cc) cartridge. The cartridge was subsequently washed with 10 mL of sterile water, and the isolated [^11^C]3 was eluted with 1 mL of ethanol into a sterile vial containing 9 mL of sterile saline. The formulated product was then filtered through a Millipore Millex^®^ GV sterile filter unit (0.22 μm) for further application in PET.

#### 3.1.4. Quality Control

The radiochemical purity and stability of all three radioligands ([^11^C]3, [^18^F]1 and [^18^F]2) were determined using analytical HPLC coupled with an XBridge analytical column (C18 5 µm, 4.6 × 150 mm particle size), a Merck-Hitachi L-7100 pump, an L-7400 UV detector, and a GM tube for radioactivity detection (VWR International). A mixture of acetonitrile (25%) and HCO_2_NH_4_ (0.1 M aq. solution) (75%) with a flow rate of 2 mL/min was used as HPLC isocratic mobile phase. The HPLC liquid flow was monitored using a UV absorbance detector (λ = 254 nm) in conjunction with a radioactive detector (BETA-flow, Beckman, Fullerton, CA, USA). All three radioligands were eluted with a retention time (tR) of 4.0–4.5 min, and the total run time of the HPLC program was 9 min. The identity of the radiolabeled compound was confirmed by co-injecting the corresponding authentic reference standard.

### 3.2. In Vitro Autoradiography

Human brains without pathological findings were acquired from the National Institute of Forensic Medicine at Karolinska Institutet in Stockholm, Sweden. These brains were obtained during forensic autopsies (designated as control brain) and were processed in a manner like previously described methods [23]. In short, experimental tissues were collected from a donor who had a post-mortem interval of 12 h. The brain was removed, and its tissues were kept in −85 °C until the sectioning process. The whole brain hemispheric slices were subsequently maintained at −25 °C until the commencement of autoradiography procedures. Permissions from the Ethics Committee at Karolinska Institutet (registration no. 03-767) were received prior to conducting the experiment. The sectioning of the brain and the autoradiography experiments were performed at the Psychiatry section, Department of Neuroscience, Karolinska Institutet, Sweden.

Whole brain hemispheres were sectioned into horizontal slices with a thickness of 100 µm using a Leica cryomacrocut system. The autoradiographic procedures followed the same protocols established in previous studies conducted in our laboratory [24]. The whole-hemisphere sections of 100 micron thickness were incubated for 90 min at room temperature with their corresponding radiotracer (4 MBq/200 mL) in TRIS buffer (pH 7.4) containing sodium chloride (120 mM), potassium chloride (5 mM), calcium chloride (2 mM), and albumin (0.1% *w*/*v*). After incubation, sections were washed three times in the same buffer at room temperature for five minutes each, briefly dipped in ice-cold distilled water, dried, and then placed in the phosphor imaging plates. Binding density standards were prepared by serial dilution of the radioligand stock solution in the assay buffer. Readings were taken with the Fujifilm BAS-5000 (Fujifilm, Tokyo, Japan), while quantitative analysis was conducted with the Multi Gauge 3.2 image analysis software (Fujifilm, Tokyo, Japan). For blocking experiments, adjacent brain sections were co-incubated with LuAF66583 (0.1 µM or 10 µM).

In case of NHP brains, fresh frozen post-mortem tissue was sectioned on a cryomicrotome (Leica CM 1860 Leica, Nussloch, Germany), thaw mounted to poly-l-lysine-treated glass plates, dried at room temperature and stored at −20 °C until use. The thickness differed from human tissue (20 µm).

### 3.3. Study Design and PET Quantification in Non-Human Primates

PET measurements were led using a high-resolution research tomograph (HRRT) from Siemens Molecular Imaging (Knoxville, TN, USA). List mode data were reconstructed using the ordinary Poisson-3D-ordered subset expectation maximization (OP-3D-OSEM) algorithm, with 10 repetitions and 16 subsets while modeling the point spread function (PSF). The in-plane resolution accomplished with OP-3D-OSEM and PSF was 1.5 mm full width at half maximum (FWHM) at the center of the field of view (FOV) and 2.4 mm at 10 cm off-center [25].

The study was approved by the Animal Ethics Committee of the Swedish Animal Welfare Agency and was performed according to “Guidelines for planning, conducting and documenting experimental research” (Dnr 5.2.18-4170/14 issued on 11 April 2014) of the KI as well as the “Guide for the Care and Use of Laboratory Animals” [26]. NHPs were supplied by the Astrid Fagraeus Laboratory (AFL) of the Swedish Institute for Infectious Disease Control (SMI), Solna, Sweden. Anesthesia was brought by intramuscular injection of ketamine hydrochloride (approximately 10 mg/kg) at AFL and continued with a mixture of sevoflurane (0–8.0%), oxygen, and medical air through endotracheal intubation at the KI PET center. The NHP head was immobilized using a homemade fixation device. Body temperature was maintained with a Bair Hugger model 505 (Arizant Healthcare, Eden Prairie, MN, USA) and monitored via an esophageal thermometer. ECG, heart rate, respiratory rate, blood pressure, and oxygen saturation were continuously examined throughout the experiments. The fluid balance was retained with continuous infusion of saline.

A 6 min transmission scan was performed before the injection of the radioligand using a single Cs-137 source. List mode data was acquired continuously for 93 min following intravenous administration of the radioligand. Images were reconstructed into 38 frames with varying intervals: 10 × 9 s, 15 × 2 s, 20 × 3 s, 30 × 4 s, 1 × 4 min, 3 × 4 min, and 6 × 12 min.

For all PET measurements, radioligands were administered via intravenous injection. Regions of interest (ROIs) were manually delineated on MRI images for each NHP, for the whole brain, cerebellum, caudate, putamen, thalamus, frontal cortex, temporal cortex, amygdala, posterior cingulate cortex, anterior cingulate cortex, hippocampus, occipital cortex, parietal cortex, ventral striatum, and insula. The summed PET images over the entire duration were co-registered with the individual NHP’s MRI image. After applying the co-registration parameters to the dynamic PET data, time–activity curves were generated for each brain region for every PET measurement. The standardized uptake value (SUV) was calculated for each brain region. The total distribution volume (*V*_T_) for all three radioligands were derived using one-tissue compartment (1-TC) and two-tissue compartment (2-TC) models [27].

On two separate occasions, PET measurements were performed after intravenous administration of GEN-7915 or PFE-360 with a slow infusion in one NHP (NHP2). The infusion lasted for 2 min, starting 30 min before PET scanning. The pretreatment formulations were prepared by dissolving it in DMSO (5%), Kolliphor EL (15%), and PBS (80%), and the pH of the final formulation was approximately 4.5–5.0. An automated blood sampling system (ABSS) measured continuous radioactivity for the first 3 min following the radioligand injection, while manual blood samples were taken at 4, 15, 30, 60, and 90 min post-injection to measure the radioactivity and metabolism.

### 3.4. Radiometabolite Analysis

The technique published earlier was utilized for the analysis of radiometabolites in NHP blood plasma [22]. During the entire PET measurement, the percentage of the injected radioligand and its corresponding radioactive metabolites and radioactivity was measured with a reverse-phase HPLC system with UV absorbance detector (λ = 254 nm) and a radioactive detector. Arterial blood samples (0.7–1.5 mL) from NHP were taken at the following timestamps: 4, 15, 30, 60, and 90 min after administering the radioligand. The blood samples were centrifuged at 4000 rpm for 2 min to separate plasma. From the centrifuged plasma, the resulting plasma was withdrawn and spiked with 1.4 times its volume of acetonitrile before being spun at 6000 rpm for 4 min. The supernatant, collected after the spun at 6000 rpm, was diluted with 3 mL of water, which was then injected into the HPLC system. The HPLC system was constructed with an Agilent 1200 series binary pump (Agilent, Santa Clara, CA, USA), a Rheodyne 7725i (Thermo Scientific, Waltham, MA, USA) manual injection valve, a 5.0 mL loop, and an Oyokoken S-2493Z radiation detector (Oyokoken, Tokyo, Japan) fitted with 50 mm lead shielding, which houses the detector. Chromatographic separation of the radiometabolites from the unchanged radioligand was performed via gradient elution on a semi-preparative reverse-phase ACE 5 µm C18 HL column (250 × 10 mm) using a mobile phase of acetonitrile (A) and ammonium formate 0.1 M (B). A flow rate of 5.0 mL/min was utilized according to the program 0–6.0 min: (A/B) 40:60 → 90:10 *v*/*v*; 6.0–10.0 min: (A/B) 90:10 (*v*/*v*). The radioactive peak corresponding to the parent radioligand was then integrated from the data output of the HPLC column. The area of this peak was calculated relative to the total area detected for all radioactive compounds. To evaluate the radioactivity recovery from the system, the measurement was taken from a 2 mL aliquot of the eluate taken from the HPLC column and measured against the total injected radioactivity.

### 3.5. Plasma Protein Binding

The plasma protein binding of all three radioligands was determined in duplicate at the baseline condition according to a previously published method. For each blood plasma sample, 500 μL of non-human primate (NHP) blood plasma was used with 50 μL PBS (pH 7.4 containing KCl 0.2 mg, KH_2_PO_4_ 0.2 mg, Na_2_HPO_4_ 1.42 mg, and NaCl 8 mg per mL). The mixture included around 20 MBq of the corresponding radioligand in PBS. Samples underwent incubation at room temperature for 10 min. A small aliquot (20 µL) was taken from each mixture, and radioactivity measurements were performed using a well counter. From each mixture, 200 µL was placed into ultrafiltration tubes (Millipore Centrifree YM-30) and centrifuged at 3000 rpm for 15 minutes. The remaining filtrates were analyzed and aliquots (20 µL) were counted in the well counter described earlier. Protein-bound fractions were calculated as follows:

(1) $$\frac{C⥂\mathrm{pla}\;(\mathrm{filtrate})\;}{C\mathrm{pla}\;(\mathrm{total})\;}÷\frac{C\mathrm{PBS}\;(\mathrm{filtrate})\;}{C\mathrm{PBS}\;(\mathrm{total})\;}\times 100(\%)$$

C_pla (total)_ represents the radioactivity concentration of incubation mixture in plasma and C_PBS (total)_ represents the radioactivity concentration of incubation mixture in PBS. C_pla (filtrate)_ and C_PBS (filtrate)_ are the radioactivity concentrations from filtrate samples.

## 4. Conclusions

Radiolabeling of [^18^F]1, [^18^F]2 and [^11^C]3 was carried out successfully. Although initial biological evaluation by in vitro autoradiography of [^18^F]1 and [^18^F]2 indicated some level of specific binding and PET imaging experiments demonstrated good brain uptake, data from in vivo PET suggests that none of these three radioligands, [^18^F]1, [^18^F]2 and [^11^C]3, are suitable for in vivo PET imaging of LRRK2.

## Figures and Tables

**Table 1:** LRRK2 wild-type (WT) and G2019S Lanthascreen, MDR1-MDCK permeability (Papp A:B), and efflux ratio (B:A/A:B), free fractions in plasma (*fp*) and brain homogenate (*fb*) and PET Multi-Parameter Optimization (MPO) score of Compounds **1**, **2**, and **3**.

Compd.	LRRK2 Lanthascreen ^1^		Papp A:B ^2^ (MDR1-MDCK) (cm/s × 10^−6^)	B:A/A:B ^2^	*fp* ^3^ (%)	*fb* ^3^ (%)	PET MPO ^4^
	WT cKi (nM)	G2019S cKi (nM)					
**1**	0.70	1.0	31	0.66	38	13	5.5
**2**	0.32	0.37	44	0.66	47	23	5.1
**3**	0.14	0.28	17	0.80	4.0	1.2	2.5

^1^ Assays described in ref. [16]; ^2^ Assays described in ref. [17], ^3^ ref. [18], ^4^ ref. [19].

**Table 2:** Body weight of NHPs, injected radioactivity, mass, and the MA at the time of administration.

NHP ID	BW (g)	Radioligand	Injected Radioactivity (MBq)	Molar Activity (GBq/µmol)	Injected Mass (µg)	Condition
NHP 1	5770	[^18^F]1	107	190	0.15	Baseline
NHP 2	6450	[^18^F]1	104	177	0.16	Baseline
NHP 2	6500	[^11^C]3	95	2804	<0.02	Baseline
NHP 1	5770	[^11^C]3	100	3500	<0.02	Baseline
NHP 3	5150	[^18^F]1	176	184	0.15	Baseline
NHP 3	4800	[^18^F]1	178	93	0.3	Pretreatment GNE-7915 (40 mg/kg)
NHP 3	5100	[^18^F]1	184	624	0.08	Pretreatment PFE-360 (0.5 mg/kg)
NHP 4	6820	[^18^F]2	128	121	0.3	Baseline
NHP 1	5750	[^18^F]2	165	163	0.3	Baseline

**Table 3:** Kinetic parameters and calculation of *V*_T_ using two different models 2-TC and 1-TC for compound [^18^F]1.

	2-TC Model			1-TC Model		
Region	Baseline	GEN-7915 (40 mg/kg)	PFE-360 (0.5 mg/kg)	Baseline	GEN-7915 (40 mg/kg)	PFE-360 (0.5 mg/kg)
CB	4.41	5.08	5.69	4.13	5.03	5.67
PUT	5.89	7.71	8.25	5.71	7.56	8.25
CAU	5.87	7.89	8.50	5.83	7.78	8.50
THA	4.95	6.18	6.68	4.80	6.05	6.68
ACC	6.04	8.71	9.39	6.03	8.50	9.28
FC	5.26	6.74	7.72	5.26	6.53	7.68
AMY	6.23	7.36	7.78	6.15	7.29	7.56

**Table 4:** Calculation of the comparison of total distribution volume (*V*_T_/*f*_p_) using two different models 2-TC and 1-TC.

	2-TC Model			1-TC Model		
Region	Baseline	GEN-7915 (40 mg/kg)	PFE-360 (0.5 mg/kg)	Baseline	GEN-7915 (40 mg/kg)	PFE-360 (0.5 mg/kg)
CB	13.0	12.5	12.4	12.2	12.3	12.3
PUT	17.3	18.9	17.9	16.8	18.6	17.9
CAU	17.3	19.4	18.5	17.2	19.1	18.5
THA	14.6	15.2	14.5	14.1	14.9	14.5
ACC	19.8	21.4	20.4	17.7	20.9	20.2
FC	15.5	16.5	16.8	15.5	16.0	16.7
AMY	18.3	18.1	16.9	18.1	17.9	16.4

## Data Availability

The data presented in this study is available on request from the corresponding author due to all the supporting data being stored at the Karolinska Institutet archive.
